# ProVIA-Kids - outcomes of an uncontrolled study on smartphone-based behaviour analysis for challenging behaviour in children with intellectual and developmental disabilities or autism spectrum disorder

**DOI:** 10.3389/fdgth.2024.1462682

**Published:** 2024-09-13

**Authors:** Rinat Meerson, Hanna Buchholz, Klaus Kammerer, Manuel Göster, Johannes Schobel, Christoph Ratz, Rüdiger Pryss, Regina Taurines, Marcel Romanos, Matthias Gamer, Julia Geissler

**Affiliations:** ^1^ Department of Child and Adolescent Psychiatry, Psychosomatics and Psychotherapy, Center of Mental Health, University Hospital of Wuerzburg, Wuerzburg, Germany; ^2^ Department of Communication, University of Vienna, Vienna, Austria; ^3^ Institute of Clinical Epidemiology and Biometry (IKEB), University of Wuerzburg, Wuerzburg, Germany; ^4^ DigiHealth Institute, Neu-Ulm University of Applied Sciences, Neu-Ulm, Germany; ^5^ Chair of Special Education IV—Education for People with Intellectual and Developmental Disabilities, University of Wuerzburg, Wuerzburg, Germany; ^6^ German Centre of Prevention Research in Mental Health, University and University Hospital Wuerzburg, Wuerzburg, Germany; ^7^ Department of Psychology, University of Wuerzburg, Wuerzburg, Germany

**Keywords:** autism spectrum disorder, challenging behaviour, behaviour analysis, mental health application, cognitive behaviour therapy, parent training, parental stress, intellectual and development disabilities

## Abstract

**Introduction:**

Challenging behaviour (CB) is a common issue among children with autism spectrum disorder or intellectual and developmental disability. Mental health applications are low-threshold cost-effective tools to address the lack of resources for caregivers. This pre-post study evaluated the feasibility and preliminary effectiveness of the smartphone app *ProVIA-Kids* using algorithm-based behaviour analysis to identify causes of CB and provide individualized practical guidance to manage and prevent CB.

**Methods:**

A total of 18 caregivers (*M* = 38.9 ± 5.0) of children with a diagnosis of autism spectrum disorder (44%), intellectual and developmental disabilities (33%) or both (22%) aged 4–11 years (*M* = 7.6 ± 1.8) were included. Assessments were performed before and after an 8-week intervention period. The primary outcome was the change in parental stress. Caregiver stress experience due to CB was also rated daily via ecological momentary assessments within the app. Secondary outcomes included the intensity of the child's CB, dysfunctional parenting, feelings of parental competency as well as caregivers' mood (rated daily in the app) and feedback on the app collected via the Mobile Application Rating Scale.

**Results:**

We observed increases in parental stress in terms of conscious feelings of incompetence. However, we also saw improvements in parental stress experience due to CB and overreactive parenting, and descriptive improvements in CB intensity and caregiver mood.

**Discussion:**

*ProVIA-Kids* pioneers behaviour analysis in a digital and automated format, with participants reporting high acceptance. Pilot results highlight the potential of the *ProVIA-Kids* app to positively influence child behaviour and caregiver mental health over a longer intervention period.

**Registration:**

The study was registered at https://www.drks.de (ID = DRKS00029039) on May 31, 2022.

## 1 Introduction

Behaviour “can be described as challenging when it is of such an intensity, frequency, or duration as to threaten the quality of life and/or the physical safety of the individual or others and it is likely to lead to responses that are restrictive, aversive or result in exclusion” (1). Challenging behaviour (CB) in children encompasses, e.g., verbal and physical aggression towards others, auto-aggression, or non-compliance (2–4).

CB poses a significant obstacle to independence, learning, community integration, socialization, and public perception for individuals with mental disorders such as autism spectrum disorder (ASD) or intellectual and developmental disabilities (IDD), thus placing a substantial strain on the individuals themselves and on their caregivers (5, 6). Furthermore, managing CB comes at a high cost and often results in a significant burden of care, for example requiring special education and training for caregivers, and involvement of specialized health services (4, 7, 8). Research has identified CB as the best predictor of parental stress (5, 9–11).

Approximately 1% of the population has an IDD (IQ < 70), ranging from mild to profound degrees of impairment in conceptual, social and practical abilities (12). ASD has a prevalence of 2.8% and core symptoms comprise difficulties in social interaction and verbal and non-verbal communication as well as limited, repetitive and stereotyped patterns of behaviour, interests and activities. Among children with ASD, approximately 38% also have a diagnosis of IDD (13). A representative study in Germany found prevalence rates of approximately 52% for CB in students with IDD (14). The risk for CB increases further when IDD is accompanied by ASD (15). In children with ASD, the prevalence of CB is even higher at around 95% (16). Numerous studies have reported positive associations between the extent of CB and caregiver variables such as parental stress, depressive symptoms, and anxiety in families of children with ASD and/or IDD (17–21).

The risk factors for CB show considerable overlap between children with ASD and IDD. CB can arise from individual and/or environmental factors. Communication impairments constitute an important *individual* risk factor (22, 23) alongside insufficient adaptive problem-solving, and self-help abilities (24), sensory hypersensitivities (4), self-stimulation (3), physical discomfort or pain (25), symptom severity (26), comorbid psychiatric disorders (27), or intense need for care (14). *Environmental* factors are, e.g., punitive parenting (28), or an inadequate residential setting (e.g., lack of respectful communication or treatment, lack of autonomy) (29). Furthermore, CB can develop and persist due to operant learning processes and reinforcement (2, 30). The bidirectional relationship of child's CB, parental stress and parenting practices is described in the transactional model by Hastings, in which CB initiates parental stress, leading to dysfunctional parenting practices, which further exacerbate CB (31).

Behavioural interventions can be effective in reducing CB in individuals with ASD and/or IDD (32, 33). A meta-analysis on 8 randomized controlled trials (RCTs) including 653 children with ASD found a moderate effect of behavioural parent-training programs on children's disruptive behaviour (34). A recent meta-analysis by Groves et al. included 42 studies on non-pharmacological and 40 studies on pharmacological interventions for CB in individuals with IDD. The authors reported overall small effects, with no differences between non-pharmacological and pharmacological interventions. However, these results should be treated with caution as there were indications of a large number of studies supporting the null hypothesis (35). A meta-analysis by Ruane and Carr found a large effect of the behavioural parent program Stepping Stones Triple P on parenting style, moderate effects on parent-reported child problems, researcher observed child behaviour and parenting satisfaction and self-efficacy, as well as small effects on parental adjustment and parental relationship in families of children with developmental disabilities (36). However, the evidence remains inconclusive due to small effect sizes and a lack of high-quality studies along with great heterogeneity of behavioural interventions in terms of, e.g., contents, specificity and delivery. It may also be important to consider different types of CB that might have specific antecedents (e.g., pain as a trigger for self-injury). More nuanced and targeted therapeutic approaches, accounting for biological factors, may be necessary (37).

Addressing the question whether guidance by a healthcare professional is required, a meta-analysis showed that self-directed (i.e., without involvement of a therapist or other healthcare provider) parenting interventions for children with externalizing problem behaviour had a large effect on reducing parent-reported externalizing behaviour and moderate effects on reducing harsh or inconsistent discipline practices and reducing self-reported lax or dismissive discipline practices (38). Additionally, self-directed interventions had a small but significant effect on reducing parental stress, and a large effect on increasing parenting efficacy. Interestingly, the study found that self-directed interventions were comparable to therapist-led interventions in improving child behaviour as perceived by parents. Thus, self-directed parenting interventions not only have the potential to improve both child behaviour and parental well-being, but also can be a viable alternative for parents who face barriers to accessing therapist-led interventions.

Given the bidirectional relationship between CB and parental stress, interventions that aim to reduce parental stress can also lead to positive changes in child's CB (39). Hence, reducing parental stress can strengthen a family's functioning and balance, which in turn, moderates the effect of CB (40). Lewallen and Neece investigated changes in social skills of 24 children with IDD after parents participated in an 8-week mindfulness-based stress reduction intervention and found that the variance in child self-control was significantly accounted for by changes in parental attachment and discipline practices (41).

Overall, the literature emphasizes the need for interventions that focus not only on managing the child's CB but also on reducing parental stress and enhancing parental well-being (42).

However, there is a considerable shortage of resources addressing caregivers of children displaying CB. Insufficient healthcare resources and limited parent-training programs hinder access to professional support for families of children with ASD and/or IDD. Long waiting times, low access to specialized services and underrepresentation of children and adolescents with IDD in clinics contribute to the challenges faced (4, 43–46).

The increasing demand for accessible and low-threshold (i.e., freely available and easily accessible without relying on the resources of the healthcare system) interventions has driven the development of a growing number of mental health applications (MHAs) for children with ASD and/or IDD and their caregivers (47). However, empirical evidence for MHAs in the context of ASD and/or IDD is highly heterogeneous (48). Kim et al. showed that out of 695 apps listed by the nonprofit organisation “autism speaks” in 2017, 95% were not supported by any clinical evidence (49). Several studies indicate beneficial effects of MHAs for ASD, with the majority targeting a range of outcomes such as social and communication skills in children (48, 50, 51). Although scarce, there are some MHAs targeting CB in individuals with ASD and/or IDD. A narrative review by Sheehan and Hassiotis highlights that digital health interventions can support individuals with IDD and CB by providing augmented communication, facilitating behaviour analysis by monitoring apps, and supporting families through online forums (52). Johnson et al. found that children with ASD who used a social script iPad app before a neuroimaging procedure (*n* = 16) exhibited less externalizing CB during the procedure and parents showed lower levels of anxiety compared to the control group (53). Another study showed that an app used by parents can be effective to reduce stereotypy in children with ASD (*n* = 7) by implementing a personalized behavioural intervention (54). The app *Smartautism* by Bonnot et al. lets caregivers of children with ASD record the child's behaviour and daily routines via ecological momentary assessments (EMAs) and displays a graphical representation of the data, but offers no guidance or recommendations. The study only explored the usability and usage intention in a prospective longitudinal exploratory open study (*n* = 65) without examining efficacy. In conclusion, MHAs hold great potential for supporting caregivers of children with ASD and/or IDD (55). However, there is a notable scarcity of evidence-based MHAs that specifically target caregivers of children displaying CB as both users and beneficiaries.

The *ProVIA* project aimed at providing an evidence-based, low-threshold tool for caregivers of children with ASD and/or IDD to improve their understanding of CB and guide them in the modification of the child's behaviour via the app *ProVIA-Kids*. *ProVIA-Kids* is the first app to automate behaviour analysis to provide individualized feedback on risk factors of CB and suitable recommendations to caregivers. Additionally, *ProVIA-Kids* emphasizes the strengthening of caregivers' resources to reduce stress and positively influence the child's behaviour. The aim of this pilot study was to investigate the feasibility and preliminary effectiveness of using the *ProVIA-Kids* app for caregivers of children with ASD and/or IDD showing challenging behaviour. We hypothesized that the use of the *ProVIA-Kids* app would reduce parental stress over an eight-week intervention period. As secondary outcomes, we expected to find improvements in terms of child's CB, dysfunctional parenting, parental mood and parental competence.

## 2 Materials and methods

### 2.1 Experimental design and recruitment

Data for this pre-post study without control group were collected before (T0) and after eight weeks of using the *ProVIA-Kids* app (T1). Follow-up data collection is ongoing. The app generated a unique code under which data were transmitted to the university. Participants provided this code to the study team, who kept a paper list containing the study ID and the corresponding app code to link data from questionnaires to data transmitted from the app. All data was encrypted before transmission to prevent unauthorised access to confidential information. Access to the patient identification list was limited to the principal investigators. The study was approved by the ethics committee of the Medical Faculty of the University of Wuerzburg, Germany (AZ 233/21-me) on May 11, 2022 and registered on May 31, 2022 at the German Clinical Trials Register (https://drks.de/search/de/trial/DRKS00029039). The project was funded by the Bavarian Ministry for Family, Labor and Social Affairs.

Participants were mainly recruited from the general outpatient clinic at the Department of Child and Adolescent Psychiatry, Psychosomatics and Psychotherapy of the University Hospital of Wuerzburg between June 2022 and November 2022. Information about the study and contact details of the study team (email address and phone number) were additionally distributed via medical and care institutions, a podcast and a press release.

### 2.2 Trial flow

Families who contacted the study team via email or phone expressing interest in participating were extensively informed about the purpose of the study as well as their rights (verbally and via written participant information documents) and were provided a consent form. They were given sufficient time to decide on their voluntary participation. After all primary caregivers had signed the consent form, families fulfilling all inclusion criteria (for an overview, see Table 1) were enrolled. Participants had the right to revoke their consent at any point without giving a reason or facing any negative consequences. At the baseline assessment (T0), participants received questionnaires and provided additional sociodemographic information in an interview (face-to-face or via phone). Participants were then given access to the smartphone app *ProVIA-Kids* and instructed to use it over a period of eight weeks. One week after the baseline interview, a brief check-in phone call was scheduled to address any potential technical issues. During the eight-week intervention period, participants were encouraged to explore different app features, recommended to complete behaviour analyses after each occurrence of CB and fill out the daily mood diary for the duration of the intervention period. Especially in the first two weeks, the participants were encouraged to primarily observe the CB and fill out the behaviour analyses to identify the most common causes of CB. After those two weeks, participants were additionally encouraged to start implementing the recommended strategies for those risk factors for CB. After the intervention (T1), participants again received questionnaires and were interviewed about changes in the child's treatments and potential side effects of the app use.

**Table 1:** Summary of eligibility criteria.

Inclusion criteria		Exclusion criteria	
Child	• Age between 3 and 11 years • Diagnosis of IDD or ASD	Child	• Severe somatic, neurological or psychiatric comorbidity • Severe deprivation • Living outside of participating caregiver's home
Caregiver	• Legal guardian of the child • Sufficient German language skills for app use • Informed consent	Caregiver	• Severe psychiatric disorder impairing participation in the study

### 2.3 Sample description

Of *N* = 23 enrolled families, 3 dropped out during the study due to personal circumstances. Two families had stated their intention to return the T1 questionnaires they filled out, however, the study team did not yet receive them. The final analysis sample comprised *N* = 18 caregivers of children with a diagnosis of ASD (44%), IDD (33%) or both (22%). For analyses of data directly transmitted via the app, the two participants with missing T1 questionnaires were included. Of all enrolled children aged 4–11 years (*M* = 7.6 ± 1.8), 66% were male and 67% were diagnosed with comorbid psychiatric disorders and 72% lived with both biological parents. Sociodemographic characteristics of all children are presented in Table 2.

**Table 2:** Sociodemographic data regarding the children (ITT sample).

	M (SD)	
Age	7.6 (1.8)	
	*n*	%
Inclusion diagnosis		
Only ASD	8	44%
Only IDD	6	33%
Both ASD and IDD	4	22%
Gender (m/f)	12/6	67/33%
Living environment		
Both biological parents	13	72%
Biological mother	3	17%
Foster parents	2	11%
Kindergarten/school		
Regular kindergarten	3	17%
Special needs kindergarten	2	11%
Special needs school	8	44%
Pre-school for special education	2	11%
Montessori school	1	6%
Secondary school	2	11%
Verbal	15	83%
Toilet-trained at daytime	12	67%
Toilet-trained at nighttime	11	61%
Physical illnesses	8	44%
Comorbid psychiatric disorders		
No comorbid diagnoses	5	28%
Attention deficit (hyperactivity) disorder	6	33%
Nonorganic insomnia	3	17%
Mixed specific developmental disorders	6	33%
Nonorganic enuresis	3	17%
Nonorganic encopresis	3	17%
Social anxiety disorder of childhood	1	6%
Overactive disorder associated with mental retardation and stereotyped movement	1	6%
Expressive language disorder	1	6%
Elective mutism	1	6%
Obsessive compulsive disorder	1	6%
CGI		
Moderately ill	1	6%
Markedly ill	6	33%
Severely ill	7	39%
Among the most extremely ill patients	4	22%

All app users (referred to as “study participants”) were female between 28 and 51 years (*M* = 38.9 ± 5.0) with 39% having a psychiatric diagnosis, and 6% having had a clinical visit due to psychiatric symptoms. Chronic illness was present in 28% of the main app users. In 44% of cases, study participants reported a higher education entrance qualification, 61% worked part-time and 11% worked full-time. All co-parents worked either full-time (94%) or part-time (6%). Sociodemographic characteristics of all caregivers are presented in Table 3.

**Table 3:** Sociodemographic data regarding study participants and co-parents (ITT sample).

	Study participants		Co-parents	
	M (SD)		M (SD)	
Age	38.8 (5.0)		42.3 (4.7)	
	*n*	%	*n*	%
Gender				
Female	18	100%	0	0
Male	0	0%	18	100%
Health				
Very good	3	17%	4	22%
Good	11	61%	8	44%
Acceptable	4	22%	3	17%
Poor	0	0%	0	0%
Very poor	0	0%	0	0%
Mother tongue				
German	16	89%	14	78%
Other	2	11%	3	17%
School qualification				
Higher education entrance qualification	8	44%	3	17%
Vocational school leaving certificate	0	0%	3	17%
Secondary school leaving certificate	4	22%	6	28%
Lower secondary school leaving certificate	5	28%	6	33%
Another school leaving certificate	1	6%	1	6%
Highest level of education				
Doctorate degree	0	0%	1	6%
University degree	4	22%	0	0%
Polytechnic degree	2	11%	1	6%
Degree from a vocational school, master school, technical school, school of health care, specialized academy	3	17%	2	11%
Degree from an upper secondary school, professional school, secondary technical or vocational school	1	6%	3	17%
Completed apprenticeship, degree from a commercial school	5	28%	9	50%
Other degree	1	6%	2	11%
Employment				
Full-time	2	11%	17	94%
Part-time	11	61%	1	6%
Unemployed	5	28%	0	0%
Chronic diseases	5	28%	6	33%
Psychiatric diagnosis				
No psychiatric diagnosis	10	56%	13	72%
First clinical presentation due to symptoms	1	6%	1	6%
Existing psychiatric diagnosis	7	39%	2	11%
Unknown	0	0%	2	11%

There was no difference between study completers and dropouts regarding initial parental stress (EBI total score; *p* = .182) with the exception of the subscale “parental attachment”, which revealed higher scores in completers than in the dropouts, *t*(21) = 2.86, *p* = .005. Among dropouts, 80% reported a psychiatric diagnosis whereas this was the case for only 40% of the study completers. For a comparison of baseline characteristics of completers and dropouts, see Supplementary Table S1.

### 2.4 Intervention

**Figure 1:**
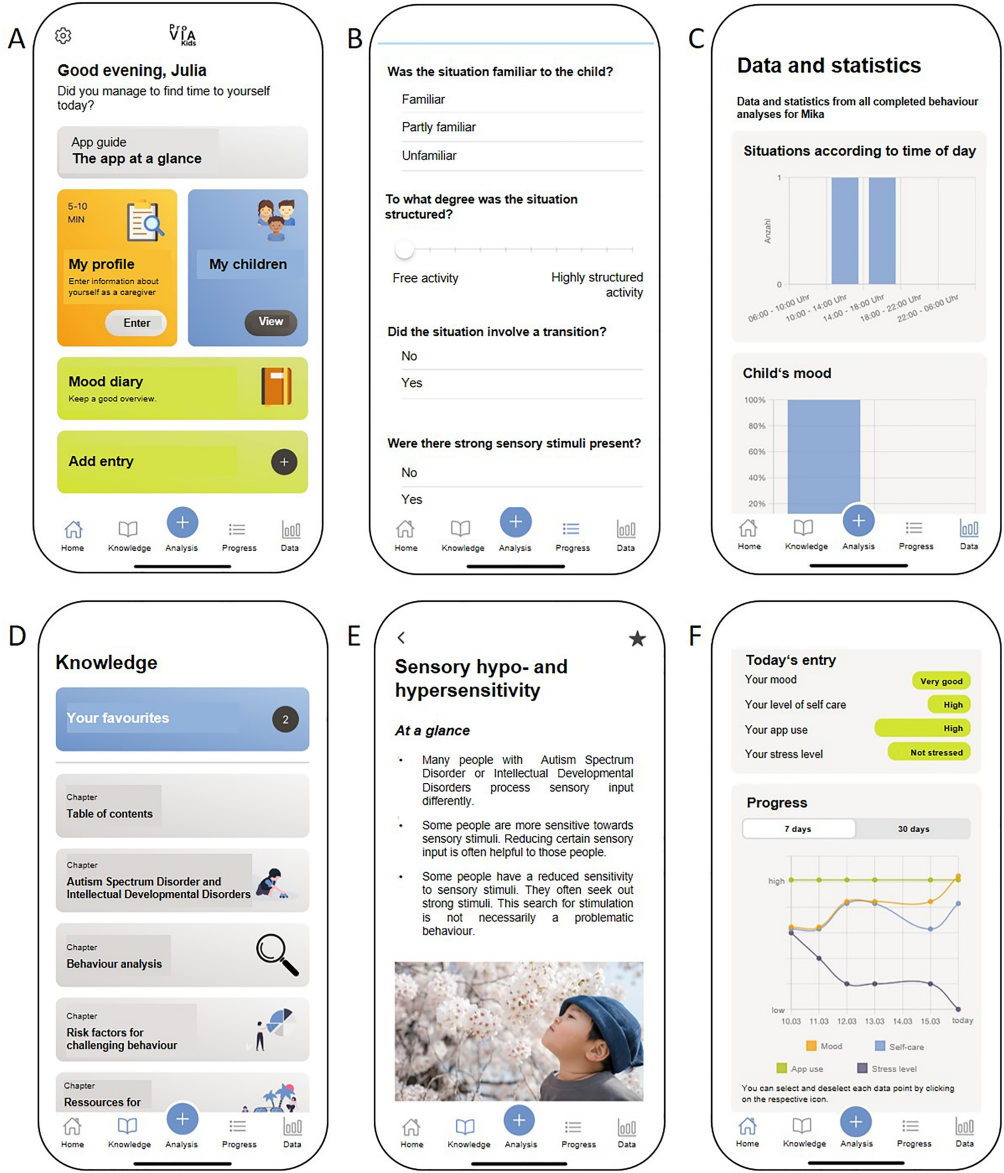
Features of the ProVIA-kids app. **(A)** Home screen including child and caregiver profiles, **(B)** behavioural analysis, **(C)** data and statistics, **(D)** psychoeducational chapters, **(E)** chapter example, **(F)** mood diary. The illustration is taken from the publication of the study protocol (56).

The *ProVIA-Kids* app was developed in cooperation with Prof. Dr. Rüdiger Pryss, Professor of Medical Informatics at the Institute for Clinical Epidemiology and Prof. Dr. Christoph Ratz, Chair of Special Education IV—Education for People with Developmental and Intellectual Disabilities at the University of Wuerzburg. *ProVIA-Kids* was developed for the Apple iOS and Android mobile operating systems. An overview of the menu items is shown in Figure 1. The app has a strong psychoeducational focus while also applying and teaching basic techniques used in cognitive behavioural therapy (CBT), such as behaviour analysis or contingency management.

*ProVIA-Kids* examines potential risk factors for CB based on a behaviour analysis algorithm and provides caregivers with appropriate recommendations, while also emphasizing strengthening caregivers' resources.

To identify *cross-situational* risk factors, users complete profiles for themselves and the child. The caregiver profile comprises 20 items related to physical needs (e.g., sufficient sleep, hydration and food intake), social support, relationship with the child, stress experience and depressive symptoms (based on the PHQ-9) (57). The child's profile comprises 69 items relating to physical and emotional needs, communications skills and sensory processing. Based on the answers, the app identifies cross-situational risk factors for each individual and provided appropriate psychoeducational information and recommendations. For example, in response to the question “Is the child”s daily schedule predictable and well-structured for the child?”, answering “no” leads to “lack of structure” being flagged as a potential risk factor and triggers recommendations for providing adequate structure.

To identify *situation-specific* risk factors, users complete a behaviour analysis after CB had occurred in a specific situation. The app asks a series of single- or multiple-choice questions regarding the nature of the situation (time of day, presence of other people, novelty/structuredness of the situation, presence of strong sensory stimuli), the child's physical and mental state (mood, pain, frustration), the consequences of the behaviour (positive, negative or both) and the regularity of these consequences (first-time, intermittent, always), thereby following the SORKC principle used in cognitive behavioural therapy (58). For each question, specific answers (e.g., “Yes, the child was frustrated.” or “The situation had a low degree of structuredness.”) are marked as pathological. Upon completion of the behaviour analysis, users receive a summary of all identified situation-specific risk factors. For each identified risk factor, the users are provided with an explanation as to why it poses a potential problem for people with ASD and/or IDD and why it can lead to CB as well as brief suggestions on how to moderate the factor and thus prevent the CB in the future. Each short recommendation contains links to psychoeducational knowledge chapters within the app with more in-depth background information about that risk factor and more detailed practical recommendations.

All conducted behaviour analyses are stored in the app, allowing users to review the data at their own pace. In addition, the risk factors identified across all behaviour analyses are aggregated and their relative frequency was illustrated graphically. This allows users to recognize and understand recurring patterns in the CB (e.g., in 75% of the situations entered, there had been a change in the child's daily routine beforehand).

Independently of behaviour analyses and profiles, users can read psychoeducational chapters on risk factors for challenging behaviour and resources for caregivers included in the app.

Finally, the app features a “mood diary”, where users can voluntarily rate their daily mood, self-care, app use and stress experience due to CB on a 6-point scale. The input is used to create a graph with four trend curves to visualize changes over time as well as interactions (e.g., stress experience decreases as self-care increases).

### 2.5 Outcomes

All outcomes were assessed at T0 and T1. A complete description can be found in the published study protocol (56). For each family, outcomes measures were completed by the caregiver who primarily participated in the intervention.

The primary outcome *parental stress* was assessed with the “Eltern-Belastungs-Inventar” (EBI (59), the German version of the “Parenting Stress Index” (60) with 48 items using a 5-point Likert scale (1 = “does not apply at all” to 5 = “fully applies”). Higher scores represent greater parental stress. The EBI differentiates between two main sources of parental stress: impairment in parental function and child's characteristics and behaviour. The parenting domain contains seven subscales (parental attachment, isolation, competence, depression, health, role restriction, spouse; e.g., “Sometimes I find it difficult to empathize with my child.”, “In order to meet my child's needs, I have to restrict myself more than I had expected.”) and the child domain contains five subscales (distractibility/hyperactivity, acceptability, demandingness, adaptability, and mood; e.g., “My child does several things that bother me.”, “My child sometimes has difficulties adjusting to changes in the daily routine or home environment.”). The EBI shows excellent internal consistency for the total scale (Cronbach's Alpha, *α* = 0.95), the parent subscale (*α* = 0.93) and the child subscale (*α* = 0.91).

As secondary outcomes, all participants were prompted once daily at a prespecified time by the app to indicate their parental stress due to CB (using 6-point scale with the anchors “not stressed at all” to “highly stressed”) and parental mood (using 6-point scale with the anchors “very bad” to “very good”), assessed via EMA. Participants could only fill out the mood diary once per day. It was not possible to retrospectively fill out the mood diary for previous days.

Additional secondary outcomes were changes in the intensity of the child's CB (assessed on a self-constructed 5-point scale), experienced parenting competence (assessed with the “Fragebogen zum Kompetenzgefühl von Eltern” (61)) and dysfunctional parental practices (assessed with the short form of the “Erziehungsfragebogen” (62)). The technical aspects of the app were evaluated using the user version of the Mobile Application Rating Scale (uMARS).

### 2.6 Data analysis

For the statistical analysis IBM SPSS Statistics 28.0 (IBM Corp., 2020) was used. The significance level was set to *α* = .05 and in order not to lose statistical power, no adjustment for multiple testing was applied. To examine pre-post changes (T0 to T1) in parental stress, CB intensity and parenting practices, paired sample *t*-tests or nonparametric Wilcoxon sign-rank tests or sign tests were performed as applicable. Pre-post-changes in caregivers' mood and stress experience due to CB were evaluated using a paired sample *t*-test. Correlations (Pearson's *r*) were calculated to examine the relationship between the change in caregiver's mood (difference in pre- and post-scores of caregiver's mood, calculated by subtracting the pre-score from the post-score) and the change in the caregiver's stress experience due to CB (difference in pre- and post-scores of stress experience, calculated by subtracting the pre-score from the post-score). Effect sizes were calculated with Cohen's *d* for mean-based analyses or Eta-squared (*η*^2^) for median-based analyses. Per protocol (PP) and intention-to-treat (ITT) analyses were conducted for the pre-post changes in parental stress, CB intensity and parenting practices. The primary analysis is based on ITT, where all participants are included in the analysis regardless of whether they completed the intervention as instructed (i.e., no minimum usage of the *ProVIA-Kids* app). PP analyses only included participants who (1) provided at least 15 behaviour analyses or mood diary entries and (2) used the mood diary for at least six weeks with at least one entry per week. To identify potential factors leading to dropouts, differences in initial parental stress and CB between study completers and dropouts were calculated using independent *t*-tests or nonparametric Mann-Whitney *U*-tests, as applicable. Differences in single parenthood were calculated using Fisher's exact test and differences in employment and psychiatric diagnoses were calculated using Fisher-Freeman-Halton exact tests.

## 3 Results

### 3.1 User satisfaction with app quality

**Figure 2:**
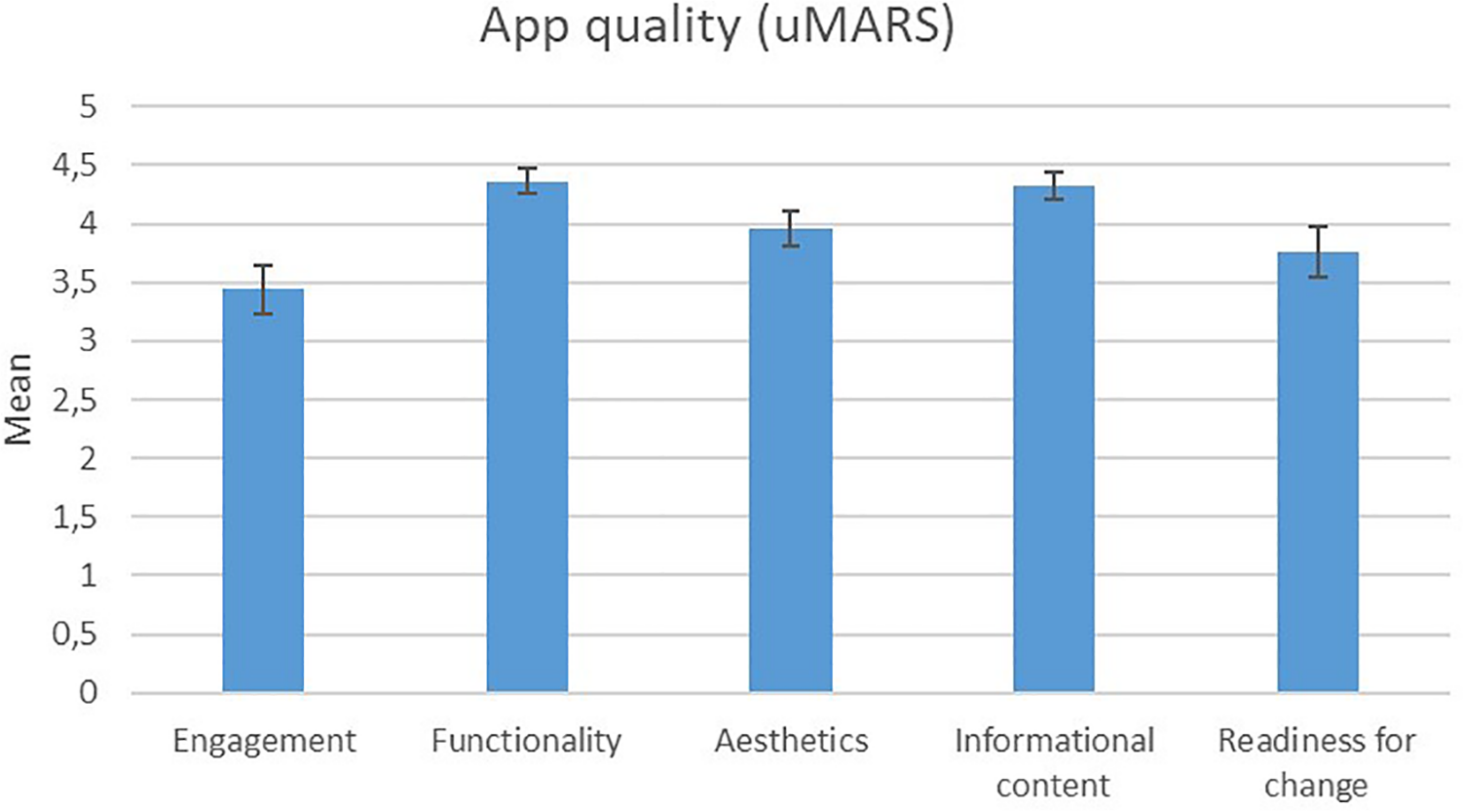
Ratings regarding app quality with the uMARS questionnaire.

Mean treatment satisfaction (1 = “strongly disagree” to 5 = “strongly agree”) was *M* = 3.1 ± 0.8. The quality of the app (Figure 2) was rated as high in terms of functionality (*M* = 4.4 ± 0.1), aesthetics (*M* = 4.0 ± 0.2) and information content (*M* = 4.3 ± 0.1) and average in terms of engagement (*M* = 3.4 ± 0.2). The willingness to change health behaviour (*M* = 3.8 ± 0.2) resulting from app use was also high.

### 3.2 Evaluation of the knowledge chapters

We received 113 ratings on 37 knowledge chapters from study participants via the app. For an overview of the ratings regarding overall article quality, comprehensibility of the information, usefulness of the recommendations, time taken to read knowledge articles, frequency of applying the recommendations in everyday life and readiness to change health behaviour, see Table 4.

**Table 4:** Feedback for the knowledge chapters (*n* = 113 ratings).

	M ± SD
How do you rate this article overall? (1 = very bad, 6 = very good)	5.0 ± 1.1
Was the information easy to understand? (1 = not easy to understand, 6 = very easy to understand)	5.7 ± 0.8
How much time did you take for the article? (1 = very little, 6 = very much)	4.4 ± 0.9
How helpful were the recommendations for you and the child? (1 = not helpful at all, 6 = very helpful)	4.0 ± 1.6
How do you rate the extent of recommendations? (1 = too short, 6 = too long)	
1	*n* = 12
2	*n* = 4
3	*n* = 26
4	*n* = 47
5	*n* = 22
6	*n* = 3
How often have you used the recommendations in your daily life? (1 = never, 6 = very often)	2.9 ± 1.6

### 3.3 Frequency of identified causal factors for CB

A total of 213 behaviour analyses were conducted with the *ProVIA-Kids* app. On average, 10.7 ± 11.1 (range 1–36) behaviour analyses were carried out by the 18 participants of the ITT sample. In most cases, the caregivers were able to state that the challenging behaviour was accompanied by positive consequences for the child (73%). In 95% of the situations, at least one other person was present and in 58% of situations at least one instruction was given. Changes in the daily routine were present in 49% of the situations. The strength of the child's feeling in the situation was reported as moderately strong (*M* = 6.0 ± 1.8; scale 1–10). An overview of the frequency of all potential causal factors can be found in Table 5.

**Table 5:** Frequency of the identified causal factors across *N* = 213 behavioural analyses.

	*N*	%
Child's mood		
Positive	154	72
Negative	59	28
Frustration	105	49
Pain		
Yes	3	1
Maybe	13	6
Change in the daily routine	105	49
Unfamiliar situation		
Unfamiliar	16	8
Partially familiar	55	26
Transitional situation	75	35
Disturbing sensory stimuli	76	36
Number of people present		
No one	10	5
One person	91	43
Multiple persons	112	53
Instruction given		
At least one instruction	124	58
Multiple instructions	30	14
Consequences of the behaviour		
Positive	118	55
Negative	57	27
Positive and negative	38	18
	M ± SD	
Intensity of the child's emotion (1 = very weak, 10 = very strong)	6.0 ± 1.8	
Degree of structuredness of the situation (1 = low degree of structuredness, 10 = highly structured activity)	5.7 ± 2.5	

### 3.4 Increase in questionnaire-based parental stress

Contrary to our hypothesis, the ITT analysis showed a significant increase in total EBI scores and thus parental stress from baseline to the post-assessment, *t*(17) = −2.19, *p* = .021, *d* = 0.52. In the PP analysis, no significant difference in the total EBI scores was found, *t*(12) = −1.28, *p* = .112.

Additionally, in the ITT analysis, there was a significant effect in the parent domain with higher scores after the treatment, *t*(17) = −3.07, *p* = .003, *d* = 0.73. This effect was descriptively smaller in the PP analysis, *t*(17) = −2.14, *p* = .027, *d* = 0.59.

**Figure 3:**
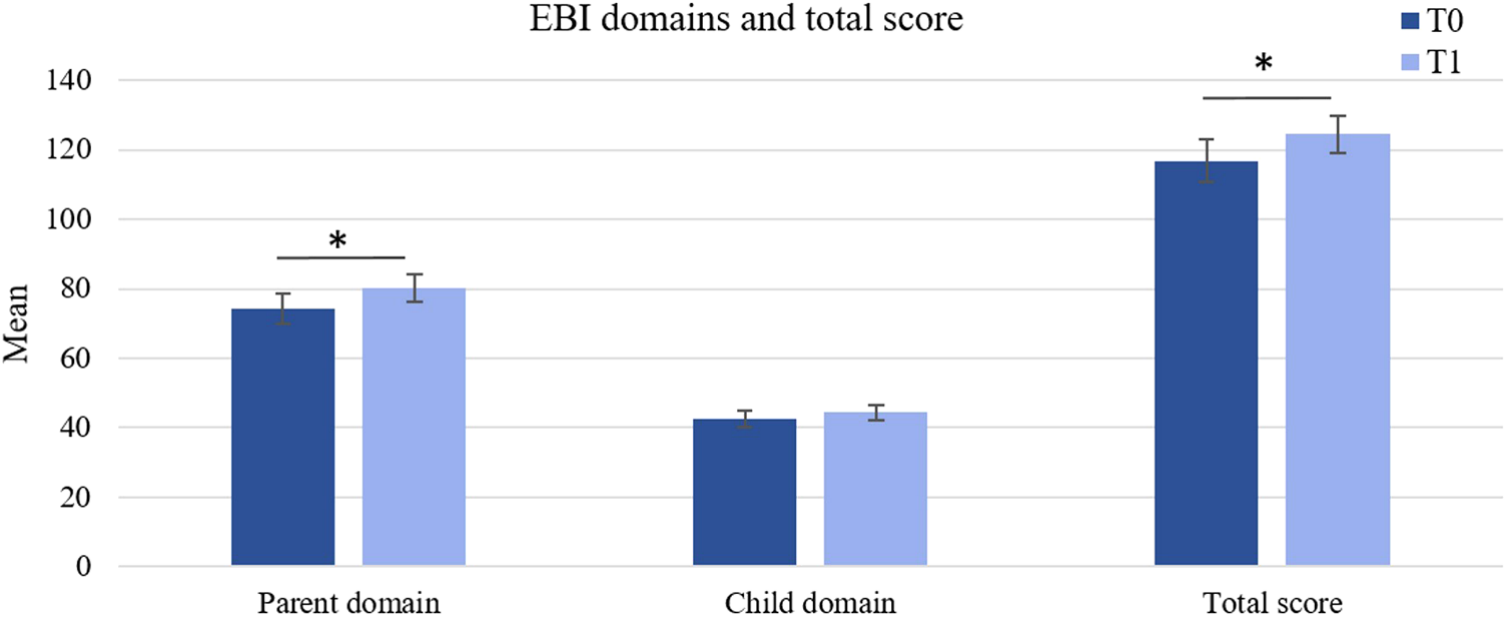
Changes in parental stress between T0 and T1 (ITT sample). Error bars represent standard errors of the mean. *p ≤ .05. **p ≤ .01.

Figure 3 shows the changes in the EBI parent and child domains and total score from pre to post treatment.

ITT analyses of EBI subscales showed that participants were scoring higher on the parent domain subscale “attachment” after the treatment, *t*(17) = −1.79, *p* = .046, *d* = 0.42. However, in the PP analysis, no significant difference in the “attachment” scores could be found (*p* = .101).

Significant increases were observed in the parent domain subscale “competence”, *t*(17) = −2.07, *p* = .027, *d* = 0.49. This effect was also observed in the PP sample, *t*(17) = −1.92, *p* = .040, *d* = 0.53.

**Figure 4:**
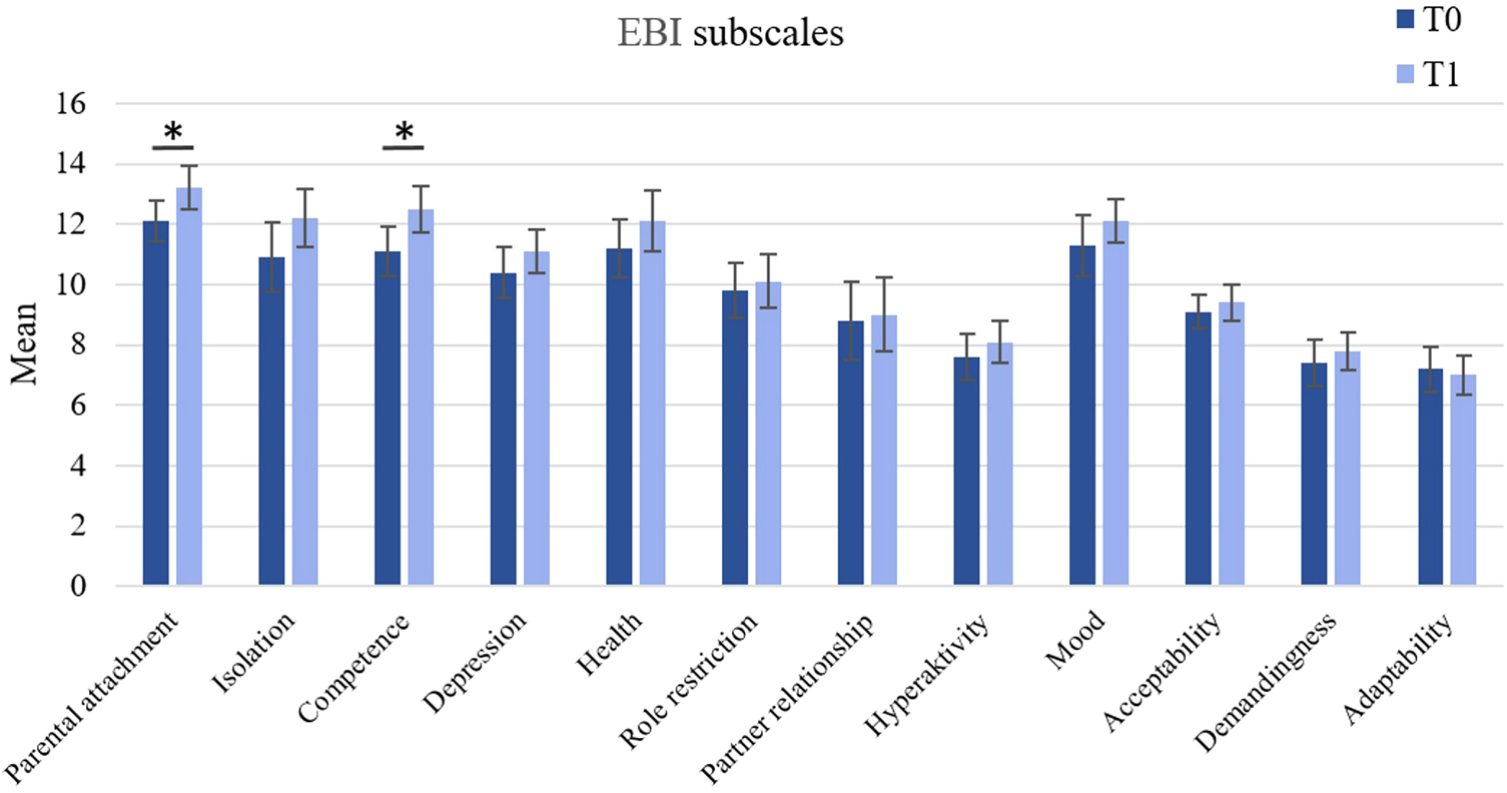
Changes in parental stress between T0 and T1 (ITT sample). Error bars represent standard errors of the mean. *p ≤ .05.

The PP analysis furthermore showed a significant increase in the parent domain “isolation” scores, *t*(17) = −1.96, *p* = .037, *d* = 0.55. Changes in the EBI subscales scores for all parent and child subscales between pre- and post-treatment are shown in Figure 4.

### 3.5 Descriptive decrease in intensity of the child's CB

**Figure 5:**
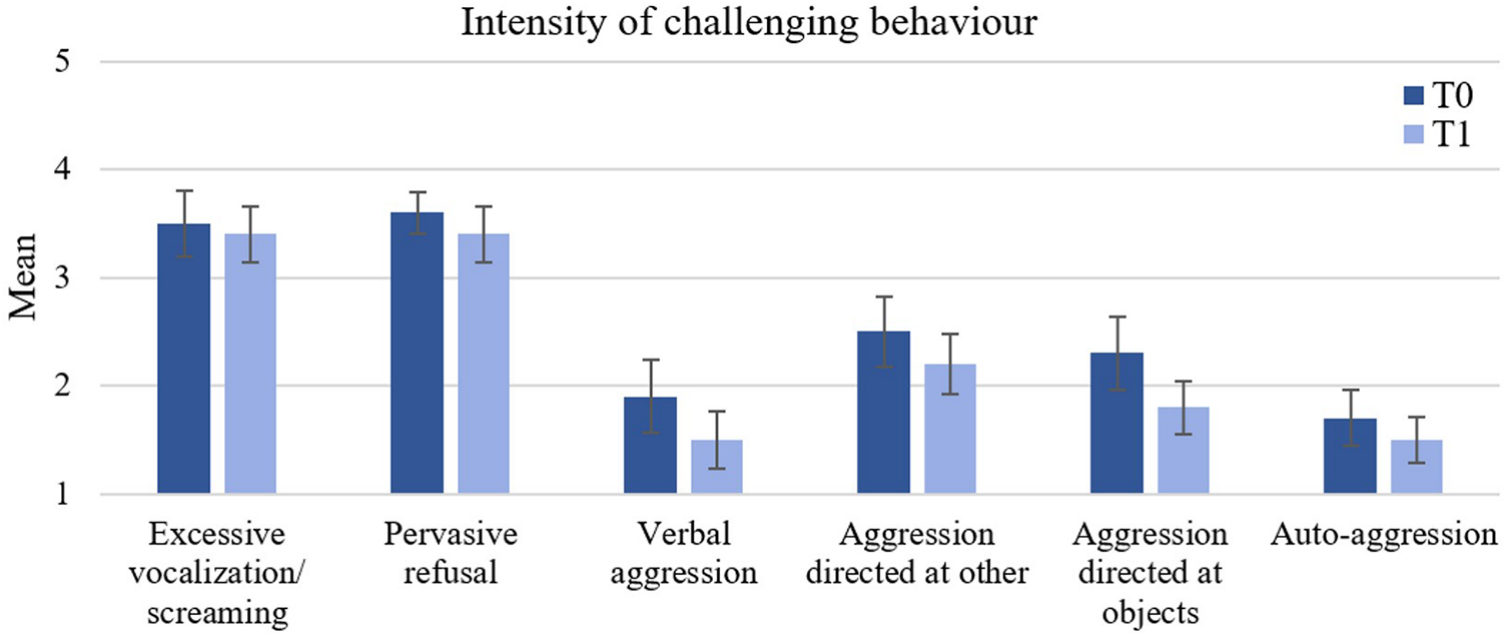
Changes in the child's challenging behaviour between T0 and T1 (ITT sample). Error bars represent standard errors of the mean.

Despite a descriptive decrease for all types of CB from T0 to T1 (as shown in Figure 5), no significant pre-post effects were found. Similarly, in the PP analyses there were no significant differences between the pre- and post-scores across all subscales.

### 3.6 Improvements in parenting practices despite decrease in subjective parental competence

There was no significant difference in total EFB-K scores before and after the treatment, *t*(16) = 1.37, *p* = .096, *d* = 0.33. However, participants scored significantly lower on the “overreactivity” subscale following the intervention, *t*(16) = 1.92, *p* = .036, *d* = 0.47. There was no significant difference in the “laxness” scores, *z* = −0.68, *p* = .495, *η^2^* = 0.03. In contrast to the ITT analyses, the PP analyses showed no significant effects.

Regarding parental feeling of competence, there was a significant decrease in total FKE scores, *t*(167) = 3.72, *p* < .001, *d* = 0.88, carried by a decrease in self–efficacy, *t*(17) = 5.79, *p* < .001, *d* = 1.37.

### 3.7 Decrease in EMA-based parental stress and descriptive improvement of parental mood

**Figure 6:**
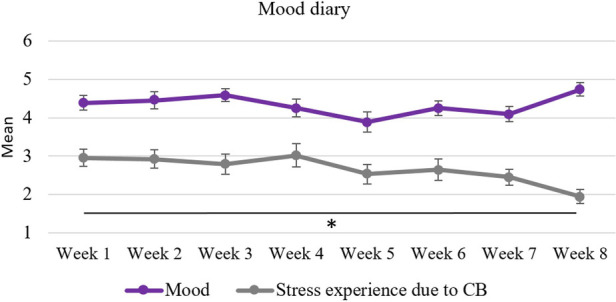
Caregiver's mood diary over the course of 8 weeks: changes in mood and stress experience due to challenging behaviour. Error bars represent standard errors of the mean. All available data sets were included which resulted in a sample size up to *N* = 20. **p ≤ .01.

EMA-based parental stress and mood were analysed for participants who had used the mood diary for at least six weeks, had made at minimum one entry per week and had available values in week 1 and week 8, resulting in a sample size of *N* = 13 (Figure 6). To examine whether the mean scores between week 1 and week 8 differed significantly, paired *t*-tests were conducted.

A statistically significant decrease in the EMA-based subjective stress experience due to CB between week 1 (*M* = 2.8 ± 0.9) and week 8 (*M* = 2.0 ± 0.8) was observed, *t*(12) = 3.29, *p* = .003, *d* = 0.91. Descriptively, a slight non-significant increase in mood scores can be seen between week 1 (M = 4.5 ± 0.9) and week 8 (M = 4.6 ± 0.7) with fluctuations in scores over time (*p* = .313).

There was no significant correlation between the change in parental mood and the change in the parental stress experience due to CB (*r* = −.381, *p* = .199). The correlations between the change in app-use and the changes in self-care (*r* = .034, *p* = .912), parental mood (*r* = .117, *p* = .705) and parental stress experience due to CB (*r* = .011, *p* = .972) were not significant. However, there was a significant correlation between change in parental mood and change in self-care (*r* = .593, *p* = .032).

For an overview of the ITT and PP results for all primary and secondary outcomes, please refer to Table 6.

**Table 6:** Primary and exploratory outcomes at baseline (T0) and after treatment (T1) divided by intention-to-treat (ITT) and per-protocol (PP) analysis.

	ITT (*N* = 18)			PP (*N* = 13)		
	T0	T1	Statistics	T0	T1	Statistics
	*M/Mdn*^a^ *(SD)*			*M/Mdn*^a^ *(SD)*		
EBI						
Parental attachment	12.1 (2.9)	13.2 (3.0)	*t* = −1.79, ***p* = .046**, *d* = 0.42	12.1 (3.1)	13.1 (3.5)	*t* = −1.35, *p* = .101, *d* = 0.37
Isolation	11.0^a^ (4.9)	12.5^a^ (4.1)	*z* = 1.25, *p* = .210, *η^2^* = 0.09	11.4 (4.3)	12.5 (3.7)	*t* = −1.96, ***p* = .037**, *d* = 0.55
Competence	11.1 (3.5)	12.5 (3.3)	*t* = −2.07**, *p* = .027**, *d* = 0.49	11.0 (3.1)	12.3 (3.7)	*t* = −1.92, ***p* = .040**, *d* = 0.53
Depression	10.4 (3.6)	11.1 (3.1)	*t* = −1.21, *p* = .122, *d* = 0.29	10.9 (3.9)	11.6 (3.2)	*t* = −1.20, *p* = .127, *d* = 0.33
Health	12.5^a^ (4.1)	11.0^a^ (4.2)	*z* = 0.97, *p* = .332, *η^2^* = 0.05	11.9 (4.2)	12.5 (4.0)	*t* = −0.97, *p* = .176, *d* = 0.27
Role restriction	10.0^a^ (3.9)	9.0^a^ (3.8)	*z* = 0.52, *p* = .607, *η^2^* = 0.02	10.0^a^ (3.3)	9.0^a^ (3.2)	*z* = 0.00, *p* = 1.000, *η^2^* = 0.00
Spouse	8.8 (5.5)	9.0 (5.2)	*t* = −0.48, *p* = .321, *d* = 0.11	9.0 (6.1)	8.9 (5.5)	*t* = 0.14, *p* = .444, *d* = 0.04
Distractibility/hyperactivity	7.6 (3.3)	8.1 (2.9)	*t* = −1.49, *p* = .077, *d* = 0.35	8.5 (3.5)	8.5 (3.3)	*t* = 0.23, *p* = .410, *d* = 0.07
Mood	11.3 (4.3)	12.1 (3.1)	*t* = −0.94. *p* = .181, *d* = 0.22	11.8 (4.5)	12.2 (3.1)	*t* = −0.37, *p* = .361, *d* = 0.10
Acceptability	9.1 (2.3)	9.4 (2.5)	*t* = −0.43, *p* = .335, *d* = 0.10	9.2 (1.4)	9.2 (2.6)	*t* = 0.10, *p* = .460, *d* = 0.03
Demandingness	7.4 (3.3)	7.8 (2.6)	*t* = −0.94, *p* = .180, *d* = 0.22	8.0 (3.4)	8.0 (2.3)	*t* = 0.00, *p* = .500, *d* = 0.00
Adaptability	5.5^a^ (3.2)	7.0^a^ (2.7)	*z* = 0.00, *p* = 1.000, *η^2^* = 0.00	5.0^a^ (3.4)	7.0^a^ (2.7)	*z* = 0.62, *p* = .537, *η^2^* = 0.11
Parent domain	74.3 (18.6)	80.1 (16.7)	*t* = −3.07, ***p* = .003**, *d* = 0.73	76.5 (19.5)	81.3 (17.8)	*t* = −2.14, ***p* = .027**, *d* = 0.59
Child domain	42.6 (9.9)	44.4 (8.9)	*t* = −0.94, *p* = .181, *d* = 0.22	44.6 (9.9)	44.6 (9.5)	*t* = 0.00, *p* = .500, *d* = 0.00
Total score	116.9 (26.5)	124.5 (23.2)	*t* = −2.19, ***p* =** **.021**, *d* = 0.52	121.1 (27.2)	125.9 (24.2)	*t* = −1.28, *p* = .112, *d* = 0.36
Challenging behaviour						
Excessive vocalization/screaming	4.0^a^ (1.3)	3.5^a^ (1.1)	*z* = −0.55, *p* = .586, *η^2^* = 0.07	3.0^a^ (1.4)	3.0^a^ (1.2)	*z* = 2.72, *p* = .414, *η^2^* = 0.57
Pervasive refusal	3.6 (0.8)	3.4 (1.1)	*t* = 0.42, *p* = .341, *d* = 0.10	3.8 (0.7)	3.5 (1.2)	*t* = 0.67, *p* = .257, *d* = 0.19
Verbal aggression	1.0^a^ (1.4)	1.0^a^ (1.1)	*z* = −0.50, *p* = .625, *η*^2^ = 0.01	1.0^a^ (1.3)	1.0^a^ (0.8)	*z* = −0.27, *p* = .785, *η^2^* = 0.02
Aggression directed at others	2.0^a^ (1.4)	2.0^a^ (1.2)	*z* = −0.32, *p* = .754, *η*^2^ = 0.01	2.0^a^ (1.6)	2.0^a^ (1.3)	*z* = −0.29, p = .774, *η*^2^ = 0.01
Aggression directed at objects	2.0^a^ (1.4)	1.0^a^ (1.0)	*z* = −1.71, *p* = .087, *η^2^* = 0.17	2.3 (1.4)	2.0 (1.0)	*t* = 0.76, *p* = .231, *d* = 0.22
Auto-aggression	1.0^a^ (1.1)	1.0^a^ (0.9)	*z* = −0.50, *p* = .625, *η^2^* = 0.01	1.0^a^ (1.2)	1.0^a^ (1.0)	*z* = −0.82, *p* = .414, *η^2^* = 0.05
EFB-K						
Laxness	2.6^a^ (0.8)	2.7^a^ (0.7)	*z* = −0.68, *p* = .495, *η^2^* = 0.03	2.8 (0.9)	2.8 (0.7)	*t* = −0.09, *p* = .466, *d* = 0.02
Overreactivity	3.6 (1.0)	3.2 (1.1)	*t* = 1.92, ***p* = .036**, *d* = 0.47	3.3 (0.9)	3.1 (1.0)	*t* = 1.41, *p* = .093, *d* = 0.39
Total score	3.1 (0.7)	2.9 (0.7)	*t* = 1.37, *p* = .095, *d* = 0.33	3.2^a^ (0.7)	2.8^a^ (0.6)	*z* = −0.18, *p* = .861, *η^2^* = 0.00
FKE						
Satisfaction	37.5 (7.6)	38.1 (8.0)	*t* = −0.53, *p* = .301, *d* = 0.13	38.9 (7.3)	39.1 (7.0)	*t* = −0.11, *p* = .456, *d* = 0.03
Self-efficacy	28.9 (4.4)	18.9 (4.0)	*t* = 5.79, ***p* < .001**, *d* = 1.37	28.9 (4.1)	19.3 (2.7)	*t* = 6.41, ***p* = <.001**, *d* = 1.78
Total score	66.4 (10.6)	57.0 (9.8)	*t* = 3.72, ***p* < .001**, *d* = .88	67.9 (10.2)	58.4 (8.4)	*t* = 3.8, ***p* = .001**, *d* = 1.05

Significant pre-post changes are highlighted in **bold**. *p*-values derived from a Wilcoxon sign-rank or sign test are two-sided. In the ITT analysis, the CB scores of verbal aggression, aggression directed at objects and EFB-K scores are based on data from *N* = 17. In the PP analysis, the CB scores of aggression directed at objects are based on data from *N* = 12.

^a^ Median *(Mdn)* instead of mean scores *(M)* are given if a Wilcoxon sign-rank test or sign test were conducted. SD, standard deviation.

## 4 Discussion

*ProVIA* investigated the feasibility and preliminary effectiveness of an 8-week CBT-based smartphone intervention with a strong psychoeducational focus for caregivers of children with ASD and/or IDD showing challenging behaviour. We expected to see reductions in parental stress, the intensity of child's CB and dysfunctional parenting practices, and an increase of parental mood and parental feeling of competence from pre- to post-intervention. The results of the study revealed a high level of acceptance and mixed effects of the intervention on the quantitative outcomes.

Regarding the feasibility of targeting CB via a smartphone application, users reported high satisfaction with the *ProVIA-Kids* app, especially in terms of information content, functionality and psychoeducational chapters. Only very few minor technical difficulties were reported, none of which interfered substantially with app usage. The positive feedback underscores not only the suitability of the app for addressing the targeted behavioural issues but also indicates a demand for this accessible, free tool among a high-need under-supported group of patients. While user engagement as measured by the uMARS was rated as average, it is worth noting that this scale primarily focuses on entertainment value and interactivity. These dimensions will be expanded in future iterations of the app based on extensive qualitative feedback gathered after the testing phase, ensuring that updates align with the specific needs of the target audience.

Regarding the intervention's preliminary effectiveness, we found mixed results. In contrast to our hypothesis, we saw an increase in parental stress from pre-treatment to post-treatment in the ITT analysis (EBI). Increases occurred in the parent domain in the subscales “parental attachment” and “competence”. While this finding is inconsistent with studies showing a reduction in parental stress via therapist-led parent-training programs for parents of children with ASD (63–65) or IDD (66, 67), as well as self-directed interventions for parents of children displaying CB (68, 69), other studies also reported an increase in parental stress after a 12-month therapist-led training program for parents of children with ASD (70, 71), IDD (72–74) and/or CB (75).

Furthermore, parental stress was measured heterogeneously in the aforementioned studies. While five studies used the “Parenting Stress Index (PSI)”, which was also used in our study, the remaining studies utilized the “Depression Anxiety Stress Scales (DASS)” (76), the “Parental Stress Scale (PSS)” (77) or the “Elternstressfragebogen” (78), thus making it difficult to compare the findings. DASS does not specifically focus on stress within the parenting context, but rather measures general emotional distress. Moreover, compared to our study, the studies using PSI showed lower baseline scores, suggesting that initial stress levels can moderate the efficacy of the intervention. A closer examination of the EBI subscales carrying the significant results provides a possible explanation as the increase in parental stress was driven by higher post-treatment scores in the two subscales “isolation” and “competence”. Caregivers who score high on the “competence” subscale feel uncertain about their decision-making abilities and lack confidence in solving parenting issues (example item: “Some things in raising my child are harder for me than I expected.”) (59). Thus, as the *ProVIA-Kids* app encourages caregivers to reflect on their own behaviour and try new strategies in managing and preventing CB via psychoeducational chapters and practical recommendations, this at first may have led to heightened feelings of incompetence when these strategies did not yield immediate positive outcomes. This is in line with research suggesting that parents require time to try new strategies at home and adapt them to their unique circumstances, thus becoming more confident in dealing with the child's CB over time (71). High scores on the “isolation” subscale, on the other hand, indicate limited integration of parents into a social network, resulting in a lack of social support and overwhelming demands of child-rearing (example item: “Since I became a mother/father, it is more difficult for me to make new contacts”) (59). However, while it is unlikely for the extent of social integration to diminish over the short period of 8 weeks, it is much more likely that the *ProVIA-Kids* app drew the caregiver's attention to their lack of social support. A further possible explanation for the results in the primary outcome could be that during the first weeks after baseline, EBI scores may have initially shown a greater increase due to the abundance of information provided by the *ProVIA-Kids* app that may at first have left the caregivers feeling overwhelmed and less competent. At T1, EBI scores may have already started to decline again but were still elevated compared to baseline. However, as there was no measurement between T0 and T1, this assumption cannot be tested. Follow-up data will shed some light on this question, if a sufficient number of participants from the small sample participates. Based on these considerations it can be concluded that future studies should include additional measurement points as well as an alternative outcome measure for examining parental stress to better understand potential changes and underlying mechanisms in the observed changes in parental stress over time.

Considering the short intervention period of 8 weeks, substantial alterations in children's CB and parenting practices were not expected. Despite a lack of significant differences between pre- and post-intervention, we could descriptively see a decrease in the intensity of all types of CB both in the ITT and PP group, indicating a potential benefit of the intervention given more time. This result is in line with previous research on parent training programs for parents of children with ASD (63, 64, 71) or IDD (66, 67, 74), which consistently revealed a reduction in CB after the intervention. This also holds true for studies on self-directed interventions for parents of children displaying CB (68, 69, 75, 79). However, it is important to note that compared to these parent interventions which last ten weeks (68), three months (65), one year (70) or even longer (64, 80), the 8-week intervention period of our study was relatively short. Moreover, research has shown a delayed effect of parent trainings on parental stress, parenting practices, and child's CB, with studies reporting significant improvements not directly after the intervention, but only 3–12 months after the intervention (65). This “sleeper effect” emerges as the techniques learned in parent trainings gradually accumulate and prove to be effective in daily life.

With regards to dysfunctional parenting practices, our study showed that while there was no significant difference in total EFB-K scores and scores in the “laxness” subscale before and after the intervention, a significant reduction was found in the “overreactivity” subscale in the ITT analysis. This pattern of findings is in agreement with prior research on parent trainings for parents of children with ASD, which consistently reported a decrease in overreactivity but no (81, 82) or a delayed follow-up (65) effect on laxness, suggesting that compared to overreactive parenting practices, lax parenting practices may be more resistant to change. However, it has to be noted that there have been studies reporting both a reduction in laxness and overreactivity after a parent training for parents of children with ASD (83) or IDD (67) or no effect on parenting practices at all (71). Thus, the significant effect on “overreactivity” may also indicate that the *ProVIA-Kids* app addressed this parenting practice more explicitly, e.g., through knowledge chapters and recommendations following behaviour analyses. The “overreactivity” subscale captures parenting mistakes such as displaying anger, irritability, or hostility, and reacting strongly emotionally in situations where calm and assertive parenting would be more appropriate [item example: “When my child is misbehaving or acting inappropriately, I raise my voice or yell at my child (vs. talk calmly to my child.)”] (61). Hence, it is possible that the intervention helped caregivers better understand the underlying reasons for their child's behaviour and manage it effectively without overreacting. Also, the “overreactivity” scores were higher than the “laxness” scores both before and after the intervention which is in line with research indicating that caregivers of children with ASD exhibit more negative parenting behaviour, including excessive control, hostility, and poor communication, than parents of typically developing children (84). However, it is important to note that at baseline the EFB-K scores in this study were below the thresholds that indicate markedly dysfunctional parenting practices (cut-offs: total score = 3.59, overreactivity = 4.34, laxness: 3.43; for an overview of mean scores of all primary and exploratory outcomes, see Supplementary Table S2).

Regarding outcomes measured via ecological momentary assessments, the hypothesis of a decline in stress experience due to CB could be confirmed. Additionally, there was a trend-level improvement in mood scores, providing hints regarding the positive impact of the intervention on parental mental health. In comparison to parental stress and dysfunctional parenting practices assessed via questionnaires once before and after the intervention, parental mood and stress experience were reported daily in the *ProVIA-Kids* app, thus avoiding reliance on retrospective reports which are prone to memory errors and biases and increasing ecological validity as data were collected in the moment of experience. It is more likely to remember information or events that are consistent with one's current emotional state (85). Generally, more weight or attention is given to negative experiences, emotions, or information, than to positive or neutral ones, even when the negative experiences may be less significant (86). Hence, while the EBI scores at T1 may not yet have decreased below the scores at T0, the stress experience measured via EMA already showed a significant decline, suggesting that participants may not have noticed the actual improvement themselves when reflecting on the entirety of the intervention period retrospectively or may have viewed the experience in a more negative light due to singular difficult situations. In conclusion, the promising results from the EMA data support the potential efficacy of the *ProVIA-Kids* app, as it offers a more nuanced understanding of caregivers' experiences. This of course needs to be verified with a randomized controlled trial.

Baseline characteristics indicate that both caregivers and children were highly burdened before the intervention. 39% of study participants had a psychiatric diagnosis and 28% were affected by a chronic illness. Most of the children were rated as severely (39%) or markedly (33%) ill and 22% were considered among the most extremely ill patients. Furthermore, parental stress scores in the subscales “parental attachment”, “isolation”, “competence”, “depression”, “health” and “mood” in the ITT sample were clinically elevated compared with normative data from a combined sample of 538 mothers (age: 20–53 years, *M* = 34.9 ± 5.5) of children aged 1–6 years (see Supplementary Table S3) (59), which is consistent with many previous studies reporting that caregivers of children with ASD and/or IDD experience more parental stress compared to caregivers of typically developing children (40). However, interpretations of the EBI scores can only be made with reservation as the normative sample comprised mothers of younger children than those included in our study. The high levels of parental stress may have impaired the caregivers' ability to engage fully in the intervention. In turn, low treatment adherence due to time constraints, lack of motivation and inconsistent implementation of learned techniques can hinder an intervention's efficacy (87, 88). Moreover, it is possible that the *ProVIA-Kids* app is more effective in supporting less burdened families whereas a more time-intensive parent program could be better suited to reduce parental stress in a more clinically affected sample (65). This consideration is further reinforced when taking into account that 80% of dropouts had an existing psychiatric diagnosis, which is roughly twice the rate of completers (39%), thus indicating that the presence of a mental disorder can pose an obstacle to study participation and probably to appropriate app use. Interestingly, completers scored significantly higher on the EBI subscale “parental attachment” in comparison to dropouts. High scores on this subscale indicate difficulties in reliably assessing the child's needs (example item: “I sometimes have a hard time figuring out what my child needs.”) (59). Consequently, a possible explanation could be that caregivers, who have greater conscious difficulties understanding and empathizing with their child experience greater distress and are therefore more willing to invest in understanding their child better through participating in the study.

## 5 Limitations and outlook

The present study has several limitations that must be taken into consideration when interpreting the results.

Firstly, the sample size was relatively small, limiting the ability to detect small to medium treatment effects due to reduced statistical power. Additionally, due to the small sample size, the study did not include subgroup analyses according to child's primary diagnosis (ASD only, IDD only, ASS + IDD), which may have provided important insights into who may benefit most from this type of intervention. Moreover, given the broad age range of the children included in this study (4–11 years), future studies should assess the efficacy of the intervention for specific age groups.

Secondly, the absence of a control group prevents attributing changes in parental and child outcomes solely to the intervention as other factors, such as, e.g., natural variations or child maturation, could also contribute to the changes. It would be valuable to further investigate whether especially caregivers with limited access to professional resources and less familiarity with their child's diagnosis experience greater benefits from the intervention. Thus, future studies should conduct an RCT with a larger sample size to allow for more robust and nuanced analyses of efficacy including moderators and mediators.

Thirdly, due to the unpredictability of the frequency of CB episodes and the number of identified causal factors for CB for each participant, it was not possible to prescribe a set treatment dosage (e.g., how often behavioural analyses should be performed, how many techniques should be put into practice, how much time should be spent reading knowledge chapters etc.), which may have led to considerable heterogeneity in terms of treatment intensity.

Furthermore, it was not monitored whether participants were able to successfully perform behaviour analyses and implement the rather complex recommendations. A therapist-led introduction to the *ProVIA-Kids* app and joint implementation of the first few behavioural analyses may increase caregivers' understanding of the rationale of behavioural analyses and enhance their ability to accurately interpret the results. Therefore, future studies should include an intervention group involving (initial) therapist guidance to assess whether this can significantly increase the efficacy of the intervention.

For the EMA-based analyses, we set very liberal criteria for who was included (used the mood diary for at least six weeks, had made at minimum one entry per week and had available values in week 1 and week 8). In a study investigating efficacy, it would be necessary to define rules regarding missing assessments and set the threshold higher than in our pilot study.

Another limitation concerns the lack of representation of male caregivers in our sample. Although families could freely choose which caregiver primarily used the app, only female caregivers enrolled as study participants. Meta-analyses show that including fathers in parent training significantly enhances positive changes in children's behaviour and parenting practices (89), although fathers are consistently underrepresented in parenting interventions (90). Future studies should strive to engage both caregivers as app users and investigate the efficacy of the *ProVIA-Kids* app separately by sex of the caregivers.

A further limitation of the study is the dependence on caregiver report to evaluate both parental stress and parenting practices as well as children's CB. Relying on a single source of information can lead to biases and inaccuracies in the collected data. In order to reach a deeper understanding of the outcome variables and increase validity and generalizability of the results, a multi-informant approach should be applied in future studies. Furthermore, since a single informant does not possess a comprehensive knowledge about the child's behaviour in various settings and contexts, a jointly usable version of the *ProVIA-Kids* app should be developed which aggregates data from all persons involved in the child's care. However, this, in turn, presents a challenge as all parties involved need to consistently use the app and coordinate in implementing the recommendations to achieve improvements in the child's CB.

Lastly, in order to improve treatment adherence and app engagement, interactive gamification elements could be incorporated in the app, which could not be realized in the present study due to time and monetary constraints.

## 6 Conclusion

The present study highlights the potential benefits of self-directed interventions in improving child CB and parental well-being in families of children with ASD and/or IDD. Even over a short period of 8 weeks, the *ProVIA-Kids* app shows promise for reducing overreactive parenting practices and participants report a reduction of EMA-based parental stress experience due to child's CB. Descriptive results indicate a potential benefit in terms of children's CB, which may take longer to fully manifest. Thus, the study addresses the high demand for evidence-based, accessible, cost-effective, and low-threshold tools for caregivers of children with ASD and/or IDD who display CB. Pioneering the translation of structured behaviour analysis into a digital and automated context, *ProVIA-Kids* presents a promising approach for guiding caregivers in systemically modifying the causes and thus preventing their child's CB. Future research should revise the app by incorporating gamification elements and conduct an RCT with a larger sample size and an extended intervention period with the aim to gain a deeper insight into moderators and mediators. Prospectively, *ProVIA-Kids* could be adapted for group and team settings such as kindergartens, school, and residential facilities by e.g., allowing joint use by multiple caregivers.

## Data Availability

The raw data supporting the conclusions of this article will be made available by the authors, without undue reservation.
