# Supplementary material for: ProVIA-Kids - outcomes of an uncontrolled study on smartphone-based behaviour analysis for challenging behaviour in children with intellectual and developmental disabilities or autism spectrum disorder
Source: Front Digit Health. 2024 Sep 13;6:1462682. doi: 10.3389/fdgth.2024.1462682 (PMC11440517; doi:10.3389/fdgth.2024.1462682)
Supplement: Supplementary file 1 [file Table1.docx]

| **Table S1:** *Baseline characteristics of completers and dropouts (regarding the main app user)* | | | | |
| --- | --- | --- | --- | --- |
|  | Completers  (*N* = 18) | Dropouts  (*N* = 5) | *t*-value/  *U*-value | *p* |
|  | *M (SD)* | *M (SD)* |  |  |
| EBI |  |  |  |  |
| Parental attachment | 12.1 (2.9) | 7.4 (4.5) | *t* = 2.86 | ***p* = .005** |
| Isolation | 10.9 (4.9) | 10.0 (6.8) | *t* = 0.35 | *p* = .365 |
| Competence | 11.1 (3.5) | 9.4 (5.9) | *t* = 0.80 | *p* = .215 |
| Depression | 10.4 (3.6) | 9.8 (5.6) | *t* = 0.31 | *p* = .379 |
| Health | 11.2 (4.1) | 9.0 (5.9) | *t* = 0.95 | *p* = .177 |
| Role restriction | 9.8 (3.9) | 9.6 (7.2) | *t* = 0.08 | *p* = .470 |
| Spouse | 8.8 (5.5) | 9.2 (7.7) | *t* = -0.14 | *p* = .445 |
| Distractibility/hyperactivity | 7.6 (3.3) | 6.0 (4.0) | *U* = 37.00 | *p* = .285 |
| Mood | 11.3 (4.3) | 9.0 (5.8) | *t* = 1.0 | *p* = .165 |
| Acceptability | 9.1 (2.3) | 7.6 (4.6) | *t* = 1.04 | *p* = .155 |
| Demandingness | 7.4 (3.3) | 6.2 (4.3) | *U* = 42.50 | *p* = .434 |
| Adaptability | 7.2 (3.2) | 7.0 (5.5) | *t* = 0.09 | *p* = .465 |
| Parent domain | 74.3 (18.6) | 64.4 (38.6) | *t* = 0.82 | *p* = .210 |
| Child domain | 42.6 (9.9) | 35.8 (22.7) | *U* = 36.50 | *p* = .275 |
| Total score | 116.9 (26.5) | 100.2 (60.5) | *t* = 0.93 | *p* = .182 |
|  |  |  |  |  |
| Employment |  |  |  | *p* = .118 |
| Full-time | 11% | 0% |  |  |
| Part-time | 61% | 20% |  |  |
| Unemployed | 28% | 60% |  |  |
|  |  |  |  |  |
| Psychiatric diagnosis |  |  |  | *p* = .461 |
| No psychiatric diagnosis | 56% | 20% |  |  |
| First clinical presentation due to symptoms | 6% | 0% |  |  |
| Existing psychiatric diagnosis | 39% | 80% |  |  |
| Unknown | 0% | 0% |  |  |
|  |  |  |  |  |
| Both caregivers participate in parenting/care |  |  |  | *p* = .709 |
| Yes | 78% | 80% |  |  |
| No | 22% | 20% |  |  |
|  |  |  |  |  |
| Child’s challenging behaviour |  |  |  |  |
| Excessive vocalization/screaming | 3.5 (1.3) | 3.3 (1.0) | *U* = 30.00 | *p* = .353 |
| Pervasive refusal | 3.6 (0.8) | 3.0 (0.8) | *U* = 23.00 | *p* = .180 |
| Verbal aggression | 1.8 (1.4) | 2.0 (1.4) | *U* = 31.50 | *p* = .378 |
| Aggression directed at others | 2.5 (1.4) | 2.5 (1.0) | *U* = 33.50 | *p* = .424 |
| Aggression directed at object | 2.2 (1.4) | 2.0 (0.8) | *U* = 35.50 | *p* = .504 |
| Auto-aggression | 1.7 (1.1) | 2.3 (1.0) | *U* = 23.00 | *p* = .133 |
|  |  |  |  |  |
| *Note*: Significant changes between the groups are highlighted in **bold**. | | | | |

| **Table S2:** *Mean scores and standard deviations of primary and exploratory outcomes at T0 and T1* | | | | |
| --- | --- | --- | --- | --- |
|  | ITT (N = 18) | | PP (N = 13) | |
|  | T0 | T1 | T0 | T1 |
|  | *M (SD)* | | *M (SD)* | |
| EBI |  |  |  |  |
| Parental attachment | 12.1 (2.9) | 13.2 (3.0) | 12.1 (3.1) | 13.1 (3.5) |
| Isolation | 10.9 (4.9) | 12.2 (4.1) | 11.4 (4.3) | 12.5 (3.7) |
| Competence | 11.1 (3.5) | 12.5 (3.3) | 11.0 (3.1) | 12.3 (3.7) |
| Depression | 10.4 (3.6) | 11.1 (3.1) | 10.9 (3.9) | 11.6 (3.2) |
| Health | 11.2 (4.1) | 12.1 (4.2) | 11.9 (4.2) | 12.5 (4.0) |
| Role restriction | 9.8 (3.9) | 10.1 (3.8) | 10.3 (3.3) | 10.5 (3.2) |
| Spouse | 8.8 (5.5) | 9.0 (5.2) | 9.0 (6.1) | 8.9 (5.5) |
| Distractibility/hyperactivity | 7.6 (3.3) | 8.1 (2.9) | 8.5 (3.5) | 8.5 (3.3) |
| Mood | 11.3 (4.3) | 12.1 (3.1) | 11.8 (4.5) | 12.2 (3.1) |
| Acceptability | 9.1 (2.3) | 9.4 (2.5) | 9.2 (1.4) | 9.2 (2.6) |
| Demandingness | 7.4 (3.3) | 7.8 (2.6) | 8.0 (3.4) | 8.0 (2.3) |
| Adaptability | 7.2 (3.2) | 7.0 (2.6) | 7.1 (3.4) | 6.9 (2.7) |
| Parent domain | 74.3 (18.6) | 80.1 (16.7) | 76.5 (19.5) | 81.3 (17.8) |
| Child domain | 42.6 (9.9) | 44.4 (8.9) | 44.6 (9.9) | 44.6 (9.5) |
| Total score | 116.9 (26.5) | 124.5 (23.2) | 121.1 (27.2) | 125.9 (24.2) |
|  |  |  |  |  |
| Challenging behaviour |  |  |  |  |
| Excessive vocalization/screaming | 3.5 (1.3) | 3.4 (1.2) | 3.3 (1.4) | 3.2 (1.2) |
| Pervasive refusal | 3.6 (0.8) | 3.4 (1.1) | 3.8 (0.7) | 3.5 (1.2) |
| Verbal aggression | 1.9 (1.4) | 1.5 (1.1) | 1.7 (1.3) | 1.4 (0.8) |
| Aggression directed at others | 2.5 (1.4) | 2.2 (1.2) | 2.6 (1.6) | 2.5 (1.3) |
| Aggression directed at objects | 2.3 (1.4) | 1.8 (1.0) | 2.3 (1.4) | 2.0 (1.0) |
| Auto-aggression | 1.7 (1.1) | 1.5 (0.9) | 1.7 (1.2) | 1.5 (1.0) |
|  |  |  |  |  |
| EFB-K |  |  |  |  |
| Laxness | 2.8 (0.8) | 2.7 (0.7) | 2.8 (0.9) | 2.8 (0.7) |
| Overreactivity | 3.6 (1.0) | 3.2 (1.1) | 3.3 (0.9) | 3.1 (1.0) |
| Total score | 3.1 (0.7) | 2.9 (0.7) | 3.0 (0.7) | 2.9 (0.6) |
|  |  |  |  | |
| Mood diary^a^ |  |  |  | |
| Mood | 4.5 (0.9) | 4.6 (0.7) |  | |
| Stress due to CB | 2.8 (0.9) | 2.0 (0.8) |  | |
|  |  |  |  | |
| *Note*: ^a^In the analyses of mood and stress due to CB, data from all participants who had available values in week 1 and week 8 were included, resulting in a sample size of *N* = 13. No PP analysis was performed in this case since as participants were identical to the participants in the ITT analysis. | | | | |

| **Table S3:** *Stanine scores and T-scores of the EBI raw values* | | | | |
| --- | --- | --- | --- | --- |
|  | ITT | | PP | |
|  | T0 | T1 | T0 | T1 |
|  | *Stanine scores* | | | |
| EBI |  |  |  |  |
| Parental attachment | **7** | **8** | **7** | **8** |
| Isolation | **7** | **7** | **7** | **8** |
| Competence | **7** | **8** | **7** | **7** |
| Depression | 6 | **7** | **7** | **7** |
| Health | 6 | **7** | **7** | **7** |
| Role restriction | 5 | 5 | 5 | 5 |
| Spouse | 5 | 5 | 5 | 5 |
| Distractibility/hyperactivity | 5 | 5 | 5 | 5 |
| Mood | **7** | **7** | **7** | **7** |
| Acceptability | 6 | 6 | 6 | 6 |
| Demandingness | 5 | 5 | 5 | 5 |
| Adaptability | 5 | 5 | 5 | 5 |
|  | *T-scores* | | | |
| Parent domain | 57 | **60** | 58 | **60** |
| Child domain | 55 | 56 | 56 | 56 |
| Total score | 56 | 59 | 57 | **60** |
| *Note*: Norming basis is data from N = 538 mothers (age: 20-53 years, *M* = 34.9 ± 5.5) of children between 1-6 years ^57^. Scores above the cut-off (Stanine ≥ 7; T ≥ 60) are highlighted in **bold**. | | | | |
